# Antioxidant Intervention to Improve Cognition in the Aging Brain: The Example of Hydroxytyrosol and Resveratrol

**DOI:** 10.3390/ijms232415674

**Published:** 2022-12-10

**Authors:** Sergio Terracina, Carla Petrella, Silvia Francati, Marco Lucarelli, Christian Barbato, Antonio Minni, Massimo Ralli, Antonio Greco, Luigi Tarani, Marco Fiore, Giampiero Ferraguti

**Affiliations:** 1 Department of Experimental Medicine, Sapienza University of Rome, 00185 Rome, Italy; 2 Institute of Biochemistry and Cell Biology, IBBC—CNR, 00185 Rome, Italy; 3 Department of Sense Organs, Sapienza University of Rome, 00185 Rome, Italy; 4 Department of Maternal Infantile and Urological Sciences, Sapienza University of Rome, 00185 Rome, Italy

**Keywords:** aging, antioxidants, cognition, epigenetic, hydroxytyrosol, Mediterranean diet, oxidative stress, resveratrol

## Abstract

Both physiological and pathological aging processes induce brain alterations especially affecting the speed of processing, working memory, conceptual reasoning and executive functions. Many therapeutic approaches to reduce the impact of brain aging on cognitive functioning have been tested; unfortunately, there are no satisfactory results as a single therapy. As aging is partly contributed by free radical reactions, it has been proposed that exogenous antioxidants could have a positive impact on both aging and its associated manifestations. The aim of this report is to provide a summary and a subsequent review of the literature evidence on the role of antioxidants in preventing and improving cognition in the aging brain. Manipulation of endogenous cellular defense mechanisms through nutritional antioxidants or pharmacological compounds represents an innovative approach to therapeutic intervention in diseases causing brain tissue damage, such as neurodegeneration. Coherently with this notion, antioxidants, especially those derived from the Mediterranean diet such as hydroxytyrosol and resveratrol, seem to be able to delay and modulate the cognitive brain aging processes and decrease the occurrence of its effects on the brain. The potential preventive activity of antioxidants should be evaluated in long-term exposure clinical trials, using preparations with high bioavailability, able to bypass the blood-brain barrier limitation, and that are well standardized.

## 1. Introduction

It is well established that both the physiological and pathological aging processes induce brain alterations affecting, particularly, some aspects of cognition, especially the speed of processing, working memory, conceptual reasoning, and executive functions [1,2,3]. Even though the majority of the aged people retain relatively well-preserved health, this trend reflects on numerous individuals, especially those with disability and fragility [4,5,6]. However, there is significant heterogeneity among older adults in the rate of decline in some abilities, such as the measures of perceptual reasoning and processing speed [7]. More precisely, age-related brain deterioration results in a scaffolding of new compensatory networks, depending on the factors that positively and negatively influence cognition. This decline is mostly associated with a dysfunction of the pre-frontal cortex, which, being especially vulnerable, tends to atrophy prematurely while aging, causing a reorganization of brain functioning, which often occurs with hemispheric lateralization of the solicited regions with more frequent bilateral brain activation [8,9,10]. Furthermore, many pathologies, such as common comorbidities in elder people, negatively affect cognition, though further studies are required to better understand how aging plays a role and how brain structure and brain function might mediate the relationship between comorbidities and age on cognition [11,12,13].

Many therapeutic approaches (cognitive and physical training, pharmacology, etc.) have been tested in literature to reduce the impact of brain aging on cognitive functioning, but nothing has been proven to bring satisfactory results as a single therapy [4]. As aging is partly contributed by free radical reactions, it has been proposed that exogenous antioxidants should have a positive impact on both aging and its associated manifestations [14,15].

This report aims to provide a summary and a subsequent review of literature evidence on the role of antioxidants in preventing and improving cognition in the aging brain.

## 2. Aging

### 2.1. General Mechanisms

Aging is an inevitable time-dependent decline of all biological functions, driven by a genetic program and linked to an increased risk for numerous diseases [16]. It has been suggested that the activation of multiple pathways due to the altered function of quality control systems monitoring the performance of the genomic and proteomic repertoire of the cells plays a major role [17]. In 2013, a total of nine biological hallmarks of aging have been identified: genomic instability, telomere attrition, epigenetic alterations, loss of proteostasis, mitochondrial dysfunction, cellular senescence, deregulated nutrient sensing, stem cell exhaustion, and altered intercellular communication [18,19,20]. 

Actually, most chronic neurodegenerative human diseases (Alzheimer’s disease, Parkinson’s disease, amyotrophic lateral sclerosis, etc.) are inherently associated with increasing age, as cells with senescence features have been detected in both brains of elders and patients with neurodegenerative disease, where they promote dysfunction [21,22]. In fact, senescent cells are characterized by sustained cell cycle arrest and production of a distinct senescence-associated secretory phenotype, and they accumulate with age and age-related diseases throughout the body, where they actively promote tissue decay. Human aging and neurodegenerative diseases comprise a series of changes at the molecular, cellular, physiological, and functional levels [23]. 

Interestingly, among the functional alterations, the cognitive, emotional, and social deficiencies are very common and are mostly linked to brain alterations. Furthermore, among the cellular changes, a major role is played by oxidative stress alterations. Since these alterations are mostly inevitable, it has been suggested that the main objectives of medical interventions for elders and by extension to neurodegenerative disease patients should focus on maximizing the ability of an individual to function in his environment, maintaining autonomy and maximizing quality of life [23]. 

### 2.2. Brain Aging

Aging affects the brain and cognition because of multiple heterogeneous etiologies including alterations at various levels: molecular and cellular, vasculature, gross morphology, and cognition [24]. Numerous are the molecular and cellular changes brought by the aging process in the brain; these are characterized by a gradual reduction of neurophysiological functions, impaired adaptive neuroplasticity, dysregulated neuronal Ca^2+^ homeostasis, neuroinflammation, and oxidative alteration of molecules and organelles [25]. 

Brain aging-associated cellular and molecular changes are often related to neurodegenerative diseases due to increased oxidative stress, inflammation, energy metabolism disorders such as deregulated autophagy, mitochondrial dysfunction, and modifications of IGF-1, mTOR, ROS, AMPK, SIRTs, and p53 as central modulators of the metabolic control [26]. Interestingly, calorie restriction, physical exercise, and mental activities seem to be able to extend lifespan and increase nervous system resistance to age-associated neurodegenerative diseases, increasing protection against ROS generation, maintaining cellular Ca^2+^ homeostasis, and inhibiting apoptosis. 

**Figure 1:**
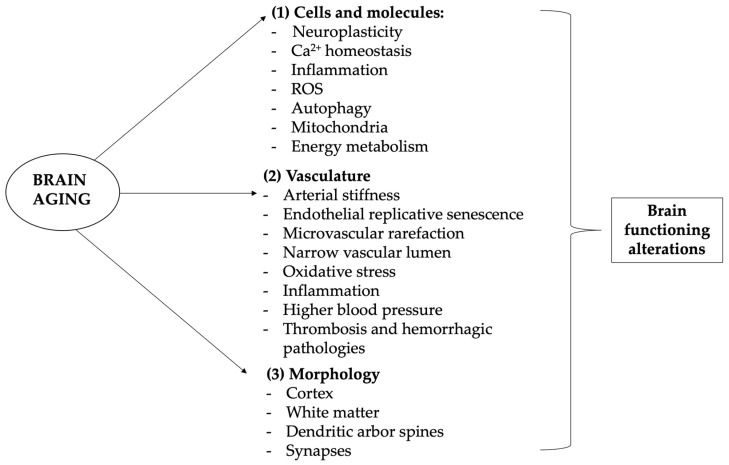
Brain-related aging mechanisms. Aging and neurodegenerative diseases are associated with cognitive, emotional, and social deficiencies mostly linked to brain alterations. Aging of the brain differs from other organs aging, as neurons are highly differentiated postmitotic cells, so that their lifespan is mostly equal to the lifespan of the entire organism. Brain aging is complex and heterogeneous but it substantially involves four levels of involvement: molecular and cellular, vasculature, gross morphology, and cognition. (1) Cellular and molecular changes involve especially (but not only) calcium-altered homeostasis, leading to hormone and neurotransmission changes, as well as ROS production, energy metabolism alteration, and neuroinflammation, which lead to progressive DNA and macromolecules damage, mitochondrial dysfunction, inflammation reaction, apoptosis, and epigenetic modifications; (2) vascular alterations and related disorders are very common and one of the leading causes of neurological disorders, morbidity, and mortality in older patients, manifesting its influence both systemically and on the more specific brain context; (3) with age come modifications of brain structure, with the frontal and pre-frontal lobes more influenced and occipital ones less affected; (4) cellular and molecular changes, but also vascular alterations and brain morphology modifications, are associated to functional impairment, which manifests mainly with memory loss and slight cognitive impairment but can lead to major pathological diseases such as dementia. In this context, antioxidants may play a major role in preventing cognitive aging problems. ROS—reactive oxygen species. Images have been created by using the functionalities of Microsoft PowerPoint 365 Version 2112. https://www.microsoft.com/microsoft-365 (accessed on 30 November 2022). Used with permission from Microsoft.

Our vascular changes and our blood pressure tend to rise, increasing the risk of stroke and ischemia and causing white matter lesions of various sizes. Aging blood vessels are characterized by shrunk blood flow, potentially leading to organ atrophy and loss of function, that in the case of cerebral vascular aging can cause loss of the blood–brain barrier integrity, eventually resulting in cognitive and sensorimotor decline as well as diseases such as vascular cognitive impairment and dementia (VCID) due to chronic cerebral hypoperfusion [27] (see Figure 1). 

Actually, multiple pathophysiological processes participate in accelerated aging and aging-related cerebrovascular disorders, including arterial stiffness, endothelial replicative senescence, microvascular rarefaction, narrowing of the vascular lumen, and oxidative stress in inflammation [28,29,30,31,32]. As we age, our brains shrink in size, particularly at the level of the prefrontal and frontal cortex and the late myelinating regions of the prefrontal and frontal lobes. All the brain is actually implicated in these alterations and, because of the individual differences observed in brain development and the aging brain, the evaluation is a complex task. Interestingly, the occipital cortex seems to be the least affected by brain aging. Brain deterioration mainly is due to the loss of neuronal cells but there are also changes in dendritic arbor, spines, and synapses. 

The brain volume and weight start declining from age 20 with age, and the rate of deterioration reaches 5% per decade after age 40 with an increased decline rate after the age of 70 [33,34]. Indeed, there is a significantly larger loss of myelin lipids than of gangliosides. The loss of myelin lipids is particularly large in the female brain after 70 years of age, while the loss in male brain seems to be linear as early as 20 years of age. 

Aging is associated with memory decline, and brain activation becomes more bilateral for memory tasks as an attempt to compensate and recruit additional networks or because specific areas are no longer easily accessible. Dementia is often associated with aging. Genetics, epigenetics, neurotransmitters, hormones, and experience all have a part to play in brain aging. Curiously, higher levels of education or occupational attainment may act as a protective factor. Healthy diet, low to moderate alcohol intake, and regular exercise are also protective. These results suggest that biological aging is not absolutely bound to chronological aging, and it may be possible to slow biological aging and even reduce the possibility of suffering from age-related diseases such as neurodegenerative pathologies.

## 3. Oxidative Stress

### 3.1. Oxidative Stress and Epigenetics

Oxidative stress or free radicals refer to an imbalance between reactive oxygen species (ROS) generation and antioxidant defense systems, which are implicated in different pathways of injury in the development of various disorders (including neurodegenerative disorders and aging) [16,35,36,37]. Interestingly, oxidative stress plays a major role in the aging process, both by direct damage and by causing epigenetic changes. Epigenetics is defined as a heritable regulation of gene expression through DNA and histone protein modifications without DNA sequence alteration [38]. Epigenetic modification is technically a reversible process, switching on/off genes in order to dynamically respond to the cellular milieu [39]. 

**Figure 2:**
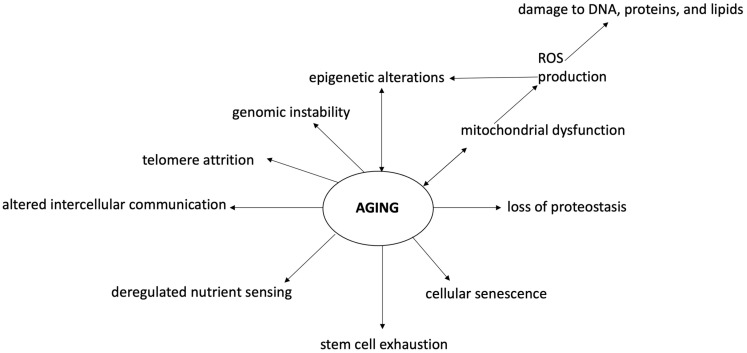
Oxidative stress and epigenetic changes in the cognitive aging process. Aging and neurodegenerative diseases are associated with cognitive, emotional, and social deficiencies mostly linked to brain alterations. Aging is characterized by a state of genomic instability, telomere attrition, epigenetic alterations, loss of proteostasis, mitochondrial dysfunction, cellular senescence, deregulated nutrient sensing, stem cell exhaustion, and altered intercellular communication. In this context, oxidative stress and epigenetic modifications play a major role, both as perpetrators and consequences of aging processes. Oxidative stress and ROS increase cause DNA and macromolecule damage associated with mitochondrial dysfunction, inflammation reaction, apoptosis, and epigenetic modifications. Epigenetic changes are associated with DNA hypomethylation, promoter hypermethylation, and altered histone modification due to various mechanisms (including oxidative stress). In this context, antioxidants may play a major role in preventing cognitive aging problems. ROS—reactive oxygen species.

Dysregulation of epigenetics is frequently found in physiological conditions such as in aging but also in almost all diseases (especially cancers) [40]. Oxidative stress and epigenetic alterations usually coincide in diseases, suggesting a close relationship between these two events (Figure 2). It has been demonstrated that ROS cause global DNA hypomethylation, promoter hypermethylation, and altered histone modification, while epigenetic regulation of ROS-mediated processes suggests the possibility of promising tools to deepen in our comprehension of the process of senescence, and to develop novel therapeutic strategies [41,42,43]. Among the highlighted tools to counter the harmful epigenetic effects of oxidative stress, dietary nutrients seem to be placed in a high spot [44,45,46]. 

### 3.2. Role of Mitochondria

Mitochondria are intracellular organelles, manned with their own circular genome (mtDNA), which play a major role in maintaining cellular homeostasis—by producing adenosine triphosphate (ATP) and intermediate metabolites—and regulating energy metabolism, cell survival and proliferation, and Ca^2+^ signaling [47,48]. Mitochondria are critical regulators of cell death, a key feature of neurodegeneration. As mutations in mitochondrial DNA and oxidative stress both contribute to aging, which is the greatest risk factor for neurodegenerative disease, the evidence suggests that mitochondria also have a central role in aging-related neurodegenerative diseases [49].

Despite being well-known hallmarks of aging, recent findings have revealed a novel crosstalk between histone epigenetic modifications and oxidative stress during stem cell aging, which once more highlights the importance of these issues for aging and age-related diseases [50]. Evidence supports the major role of mitochondrial dysfunction in promoting aging and in supporting neurodegenerative progression [51]. Mutations accumulate at a higher rate in mtDNA than in nuclear DNA, resulting in mitochondrial dysfunction and diseases, and this is even more true in older people, where there coexists a reduced mitochondrial efficiency and a deterioration of the antioxidant system [52,53,54]. 

Studies in various species highlighted several alterations in mitochondria and mitochondrial DNA (mtDNA) associated with aging: increased disorganization of mitochondrial structure; decline in mitochondrial oxidative phosphorylation function; accumulation of mtDNA mutation; increased mitochondrial production of ROS (superoxide, hydrogen peroxide, hydroxyl radicals and singlet oxygen); increased extent of oxidative damage to DNA, proteins, and lipids [55]. 

Thus, the decline in mitochondrial energy metabolism that alters quality control pathways, the enhanced mitochondrial oxidative stress, and the accumulation of mtDNA mutations are important contributors to human aging. Since the efficacy of the respiratory chain diminishes, aging is associated with electron leakage, increased ROS production and reduced cellular ATP generation [56,57]. Mitochondria have been related also to multiple diseases, often aging-related, such as neurodegeneration and cancer [58,59,60]. Interestingly, it has been suggested that novel pathways that protect the cell through mitochondrial quality control may offer unique opportunities for disease therapy in situations where ongoing mitochondrial damage occurs [61].

Some interesting studies demonstrated the major role of oxidative stress in regulating the lifespan, so reducing oxidative stress resulted in the expanded life in murine models [62,63]. In a major study, to determine the role of the reactive oxygen species in mammalian longevity and pathology, the authors generated transgenic mice that overexpress human catalase localized in the peroxisome, nucleus, or mitochondria [62]. In the mice overexpressing the human mitochondria catalase, the median and maximum lifespans were maximally increased (averages of 5 months and 5.5 months, respectively), while cardiac diseases and cataract development were delayed, oxidative damage was reduced, H_2_O_2_ production and H_2_O_2_^−^ induced aconitase inactivation were attenuated, and the development of mitochondrial deletions was reduced. 

These results support the free radical theory of aging and reinforce the importance of mitochondria as a source of these radicals [62,63]. These results have been confirmed by another study that demonstrated how the overexpression of the antioxidant enzyme catalase in mitochondria can extend mouse lifespan, highlighting the importance of mitochondrial damage in aging and suggesting that, when targeted appropriately, boosting antioxidant defenses can increase mammalian life span [63,64]. Despite the need of further studies, therapies targeting basic mitochondrial processes, such as energy metabolism or free-radical generation, or specific interactions of disease-related proteins with mitochondria, hold great promise [49].

### 3.3. Mediterranean Diet

The Mediterranean diet refers to a traditional diet consumed in Mediterranean countries and characterized by a high consumption of vegetables and olive oil and moderate consumption of food rich in proteins. It has been demonstrated that the Mediterranean diet, abundant in minimally processed plant foods, reduces the risk of developing various chronic diseases and seems to increase life expectancy [65]. In fact, this diet has beneficial effects in the primary and secondary prevention of cardiovascular disease, type 2 diabetes, atrial fibrillation, and breast cancer [66,67]. 

The exact mechanism by which an increased adherence to the Mediterranean diet exerts its favorable effects is mostly unknown; however, evidence suggests that the major role is played by: lipid-lowering effect, protection against oxidative stress, inflammation and platelet aggregation, modification of hormones and growth factors involved in the pathogenesis of cancer (reduction of DNA damages, cell proliferation, and their survival, angiogenesis, inflammations, and metastasis), inhibition of nutrient sensing pathways by specific amino acid restriction, and gut microbiota-mediated production of metabolites influencing metabolic health [66,68,69,70]. 

Interestingly, it has been demonstrated that the Mediterranean diet, and nutrients in general, can modulate gene expression directly by binding to nuclear receptors or acting indirectly modulating epigenetic effects (DNA methylation, histone modifications, microRNAs) [69,71]. Among the nutrients contained in the Mediterranean diet, olive oil and red wine are probably the most widely consumed and, when assumed at the correct dosage, they have been demonstrated to have beneficial effects on health [72,73]. In bringing these positive results, a major role is played by the presence of antioxidants in these aliments and, being among the most studied antioxidants, in the following chapters we will report the experiences on hydroxytyrosol and resveratrol [74,75,76].

### 3.4. Antioxidants

As antioxidants are substances that are efficient to trap ROS and decrease oxidative damage, these products have been studied for therapeutic approaches to many different diseases. On the other hand, a deficiency of antioxidants such as vitamins C and E has been associated with cognitive disorders [77]. In literature, many natural products and pharmacological compounds have antioxidant properties [73,78]. Interestingly, resveratrol and other polyphenols extracted from olive and wine but also other natural goods are among the most studied and possess great antioxidant and anti-inflammatory properties [79,80,81,82].

### 3.5. Polyphenols

Polyphenols are natural, synthetic, or semi-synthetic organic molecules constituted by numerous hydroxyl groups on aromatic rings (phenolic groups), presenting neuroprotective and anti-inflammatory effects and capacity of control of oxidative stress, apoptosis and mitochondrial dysfunction [81,83,84]. These products are divided into four main groups: phenolic acids, flavonoids, stilbenes, and lignans. 

The Mediterranean diet is a mainstay of nutritional therapeutic and preventive programs in many diseases because of a rich presence of foods and beverages abundant in polyphenols, such as olives, olive oil, wine, fresh and processed fruits and vegetables, leguminous plants, cereals, herbs, spices, tea, coffee, and beer [85,86]. A proper diet is one of major factors contributing to good health and is directly related to the general condition of the organism [87,88]. Polyphenols are converted and absorbed mainly in the oral cavity and stomach; in the large intestine, the remaining polyphenols are further modified by bacterial enzymes (e.g., glycosides, esters, etc.) to obtain metabolites of lower-weight easier to absorb; these metabolites then circulate within blood, bound to proteins (mainly albumin), and are conjugated in the liver and kidneys; finally, elimination happens in the urine and feces [89]. 

Polyphenols are present in liquid natural products such as olive oil and green tea; however, it is also true that they can be found in alcohols such as red wine (whose main polyphenol is resveratrol) and beer [79,81,87,90,91,92,93,94,95,96]. Interestingly, the evidence suggests that moderate wine consumption may decrease the risk of several cancers (including colon, basal cell carcinoma, ovarian, and prostate cancer) and cognitive diseases; on the other hand, it should be pinpointed to an adequate balance in order to avoid the negative effects due to the presence of substances such as ethanol [97,98,99]. 

### 3.6. Antioxidants and Cognition in the Aging Brain

Oxidative stress and the inflammation due to increased oxidative stress are associated to many chronic diseases, but the lack of anti-inflammatory drugs without side-effects has stimulated the search for new active substances. It has been demonstrated that the Central Nervous System (CNS) can benefit from nutritional strategies and dietary interventions that prevent the signs of senescence, such as cognitive decline or neurodegenerative diseases such as Alzheimer’s disease and Parkinson’s Disease [100]. Both aging and associated neurodegenerative diseases are accompanied by the decline of several brain functions, including cognitive abilities, which are related to progressive deleterious changes at biochemical and physiological levels, leading to the generation of oxidative stress, disturbed protein metabolism with accumulation of protein aggregates, mitochondrial dysfunctions, loss of synaptic connections, and ultimately neurodegeneration and cognitive decline [101]. Because of its high energy demand, the brain is more susceptible to ROS-mediated damages, as it oxidizes lipids, proteins, and nucleic acids, thereby causing an imbalance in the homeostasis, and this especially occurs in the aging brain. It has been suggested that oxidative stress is a key factor for age-associated neurodegeneration and cognitive decline due to the imbalance between the rates of production and elimination of ROS. 

Interestingly, the involvement of the heme oxygenase (HO) pathway in anti-degenerative mechanisms related to the induction of other heat shock proteins (HSPs, molecular chaperones involved in cell protection from various forms of stress) has been demonstrated during various physio-pathological conditions [102]. In fact, the vasoactive molecule carbon monoxide and the potent antioxidant bilirubin, products of the HO-catalyzed reaction, represent a protective system that is potentially active against the brain oxidative injury associated to the cognitive dysfunction in the aging-associated neurodegenerative diseases [103,104]. 

Studies on both animal and human subject demonstrated that dietary interventions and plant-derived bioactive compounds with antioxidant properties could be beneficial for recovering the memory or delaying the onset of memory impairment, especially in case of stress-mediated changes [105]. Recently, the supplementation of spice and herbs containing phenolic substances with potent antioxidant and chemo-preventive properties, such as curcumin (a powerful antioxidant derived from the curry spice turmeric), has been considered as an alternative, nutritional approach to reduce oxidant damage and neurodegenerative pathology associated with aging.

**Figure 3:**
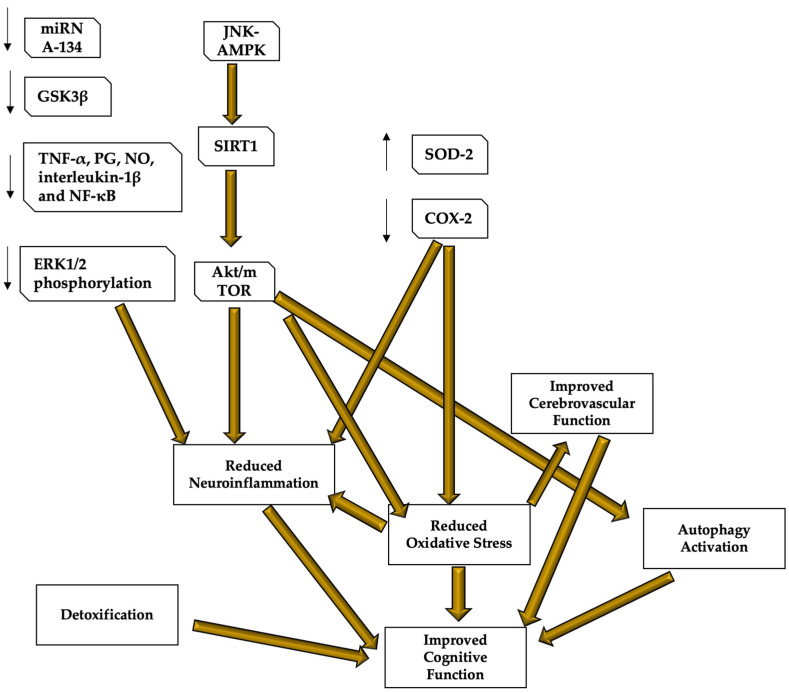
Resveratrol and hydroxytyrosol molecular mechanisms of action. Resveratrol and hydroxytyrosol demonstrated to be able to improve cognitive performance and protect the brain from neurodegenerative and age-related diseases, mostly thanks to their role in regulating oxidative stress, neuroinflammation, cerebral blood flow, and autophagy, as well as because of their capacity of detoxifying the blood from those neuro-damaging compounds. Most of the mechanisms are still under study, but it seems that both resveratrol and hydroxytyrosol target the AMPK and the subsequent pathway leading to SIRT1 and Akt/mTOR to reduce neuroinflammation and oxidative stress, and stimulating autophagy. Oxidative stress reduction also inhibits inflammatory responses and stimulates cerebrovascular function, leading to better cerebral blood flow and brain functioning. These compounds are also important detoxicants. Resveratrol mechanisms are clearer and its role in reducing neuroinflammation has been also related to the capacity of lowering mRNA134, GSK3β, ERK1/2 phosphorylation and cerebral levels of TNF-α, PG, NO, interleukin-1β and NF-κB. Resveratrol also increases SOD-2 protecting functions and inhibits COX-2 to reduce ROS production. AMPK—AMP-activated protein kinase; COX—cyclooxygenase; ERK—extracellular signal-regulated kinase; LPS—lipopolysaccharide; MMP9—matrix metalloproteinase 9; NF-κB—nuclear factor κB; NO—nitric oxide, PGES-1—prostaglandin E synthase-1; ROS—reactive oxygen species; TNF—tumor necrosis factor; SOD—superoxide dismutase. Images have been created by using the functionalities of Microsoft PowerPoint 365 Version 2112. https://www.microsoft.com/microsoft-365 (accessed on 30 November 2022). Used with permission from Microsoft.

Recently, human studies have been conducted to determine the effective nutritional and lifestyle protocols for the prevention of neurodegenerative diseases [106]. The Mediterranean diet and antioxidant and anti-inflammatory products seem to play a significant role in most of the proposed protocols [107]. Furthermore, higher adherence to the Mediterranean dietary pattern has been associated with decreased cognitive decline and incidence of neurodegenerative diseases [108]. Here, we report the example of two among the most studied antioxidant products and their role in preventing cognitive dysfunction due to brain aging: hydroxytyrosol and resveratrol (see Table 1 for the evidence on neuroprotective and cognitive role of resveratrol and hydroxytyrosol and Figure 3 for a graphic description of the resveratrol and hydroxytyrosol molecular mechanisms of action).

### 3.7. Hydroxytyrosol

As the Mediterranean diet entered the spotlight for its many beneficial properties, over the years, researchers have made an attempt to find which foods and food components are responsible for the beneficial effects on health. One of these components and currently the most actively investigated natural phenol is hydroxytyrosol, a phenolic phytochemical with antioxidant properties that can be found in olive leaves and oil [150]. 

This molecule gained particular interest for its role in various diseases due to its anti-inflammatory, antimicrobial, antiviral, antifungal, cardioprotective, neuroprotective, antitumoral, and chemo-modulating effects. The interest in this molecule has led to wide research on its biological activities, its beneficial effects on humans and in how to synthesize new molecules from hydroxytyrosol. In particular, due to its bioavailability, chemical properties and easy formulation along with its lack of toxicity, hydroxytyrosol is considered an excellent food supplement by the nutraceutical and food industries [151]. 

Furthermore, hydroxytyrosol has great pharmaceutical potential in cognitive and aging disorders because it improves endothelial dysfunction, decreases oxidative stress and has neuroprotective properties [152,153]. Unfortunately, most of the evidence comes from animal model-based experiments, but hydroxytyrosol seems to be able to stimulate neurogenesis by enhancing stem and progenitor cell proliferation and neuron survival [147].

It has been demonstrated that a diet rich in purified olive polyphenols has positive long-term effects on cognition and energy metabolism in the brains of aged mice [154,155]. Furthermore, recent studies demonstrated that hydroxytyrosol protects from the aging process by modulating the AMP-activated protein kinase (AMPK) signaling and autophagy, while the AMPK dysregulation has been associated with accelerated aging and promotion of inflammation, cancer, and metabolic pathologies such as diabetes and obesity [156]. 

Interestingly, in TgCRND8 mouse models, it has been demonstrated that hydroxytyrosol diet supplementation results in substantial neuroprotection improving cognitive functions and leading to significant changes in the brain cortex and hippocampal areas with also a marked reduction of the TNF-α expression and astrocyte reaction [144,157]. Furthermore, hydroxytyrosol is able to ameliorate neuronal impairment via modulating mitochondrial oxidative stress, neuronal inflammation, and apoptosis [121,158]. In *Caenorhabditis elegans* models, it has been demonstrated that an olive-derived extract 20% rich in hydroxytyrosol is able to prevent the β-amyloid aggregation and oxidative stress associated to Alzheimer’s disease [124,159]. These effects have been associated with the increased gene expression of the SKN-1/NRF2 transcription factor and the overexpression of HSP-16.2. In swine models, hydroxytyrosol prevented DNA hypomethylation associated with oxidative stress [160]. Interestingly, hydroxytyrosol can reduce hypoxia-mediated cell damage through activating the PI3K/AKT/mTOR-HIF-1 α signal pathway [161]. As ischemic events are more common in older people and are an important cause of neurological impairment, it is of interest noting that the hydroxytyrosol-enriched diet could serve as a beneficial therapeutic approach to attenuate ischemic stroke-associated damage [162,163]. 

### 3.8. Resveratrol

Resveratrol (3,5,4′-trihydroxy-trans-stilbene) is a natural non-flavonoid phenol and a phytoalexin with antioxidant and cytoprotective effects led by pleiotropic actions [80]. It is produced by several plants in response to injury or when these are under attack by pathogens (bacteria, fungi, etc.) and as such, it interacts with gut microbiota, inducing modifications in bacterial composition associated with beneficial effects. 

The major food sources of resveratrol are grapes, blueberries, raspberries, mulberries, and peanuts. Resveratrol has neuroprotective and antiaging properties preventing the effects related to oxidative stress, reducing DNA damage and regulating molecular changes such as mitochondrial dysfunction, inflammation reaction, apoptosis, and epigenetic modifications, among others [164,165,166,167]. 

Recent evidence demonstrated that resveratrol mediates epigenetic changes (methylation and acetylation) that persist across generations and that are involved in aging and the function of the nervous system [168,169,170,171]. The study of key markers involved in senescence and rejuvenation (mitochondrial biogenesis and Sirt1-AMPK-PGC1-α) demonstrated that resveratrol is also able to modulate the changes in these cellular metabolic pathways. Interestingly, resveratrol may exert neuroprotective effects also by regulating autophagy and apoptosis mediated by the Akt/mTOR pathway [134,135]. Unfortunately, most of the evidence on resveratrol is based on animal experiments and the effects on human cognition are likely to be smaller [94,172]. 

On the other hand, recent clinical evidence, derived from randomized clinical trials, suggests that in humans, resveratrol is able to improve cerebral blood flow, cerebral vasodilator responsiveness to hypercapnia, some cognitive tests, perceived performances, and the Aβ40 plasma and cerebrospinal fluid level, modulates neuroinflammation, and induces adaptive immunity [109,128,173,174,175]. In particular, resveratrol causes a dose-dependent increase in cerebral blood flow and enhanced oxygen extraction, but its role in ameliorating cognitive performance is still under study [127,129]. Despite the lack of definite evidence, the role of resveratrol on the cerebral blood flow is important especially considering the vascular alterations found in most of the age-related diseases [176]. Interestingly, recent findings demonstrated that cis- and trans-resveratrol have the opposite effects on histone serine-ADP-ribosylation and tyrosine-induced neurodegeneration, so that the age-associated increase in serum tyrosine levels may affect neurocognitive and metabolic disorders, offering a plausible explanation for divergent results obtained in clinical trials using resveratrol [177]. 

### 3.9. The Limitation of the Blood–Brain-Barrier

In recent years, many compounds and drugs that affect the brain have been regarded for their potential role in the therapeutic management of various age-related diseases [178,179,180]. Regrettably, the administration of therapy that is able to reach the brain has inherent problems because of the blood–brain-barrier (BBB), which prevents the brain uptake of most pharmaceuticals, so that not every product that demonstrated to be able to reduce aging progression is capable of influencing the more specific brain aging.

This BBB property comes from the epithelial-like tight junctions within the brain capillary endothelium. Providing the drug has a molecular weight under 400 Da and forms less than eight hydrogen bonds, small molecules acting as agonists, modulators or enhancers targeting the associated receptors may cross the BBB via lipid-mediated free diffusion, but these chemical properties are lacking in the majority of small molecule drugs, as well as all large molecule drugs [181]. In fact, many solutions have been suggested, for example, the protein could be infused directly, produced by viral constructs, secreted from implanted protein-secreting cells or actively transported across the brain.

Drugs can be reengineered for BBB transport: small molecule drugs can be synthesized to be able to access carrier-mediated transport (CMT) systems within the BBB; large molecule drugs can be modified using molecular Trojan horse delivery systems to access receptor-mediated transport (RMT) systems within the BBB. Peptide and antisense radiopharmaceuticals can also guarantee made brain-penetrating properties by combining the use of RMT-based delivery systems and avidin–biotin technology.

Many studies suggested that the early disruption of the BBB to large molecules is mediated by ROS [182,183]. As the brain is exposed throughout life to damages due to ROS, various endogenous antioxidant defense mechanisms physiologically exist, including the removal of O^2^, scavenging of reactive oxygen/nitrogen species or their precursors, inhibition of ROS formation, binding of metal ions needed for the catalysis of ROS generation, and up-regulation of endogenous antioxidant defenses.

However, since our endogenous antioxidant defenses are not always completely effective, and since exposure to damaging environmental factors is increasing, it has been suggested that exogenous antioxidants could be very effective in diminishing the cumulative effects of oxidative damage. Unfortunately, the therapeutic use of most of the studied antioxidant compounds is limited, since they do not cross the blood–brain barrier.

Novel antioxidant molecules designed as potential neuroprotective treatments in neurodegenerative and age-related disorders are focusing on this limitation to be able to cross the BBB after systemic administration; furthermore, gender differences have been identified [184,185]. A recent study suggested that resveratrol is well tolerated and seems to be able to penetrate the blood–brain barrier to produce its neuroprotective effects [141]. Unfortunately, most of the evidence comes from pre-clinical and animal-based studies; so, further research is required before most of these therapies can be safely applied clinically.

## 4. Discussion

This report aims to provide a summary and a subsequent review of literature evidence on the role of antioxidants in preventing and improving cognition in the aging brain. As endogenous antioxidants are linked to antidegenerative mechanisms, exogenous antioxidant supplementation could play a major role in inhibiting oxidative damage and compensating for the decreased level of endogenous antioxidants. Conceivably, dietary supplementation with antioxidant polyphenolic agents such as hydroxytyrosol, resveratrol and curcumin reduces the development of aging-related cognitive diseases, their signs, and symptoms. Among others, dietary antioxidants play a major role in preventing and reducing DNA damage, mitochondrial dysfunction, inflammation, apoptosis, and transgenerational epigenetic modifications.

These products have numerous properties with antimicrobial, antiviral, antifungal, cardioprotective, neuroprotective, antitumor, and chemo-modulating effects. These results demonstrate that numerous pathways, mostly still unknown, are concerned, with pleiotropic actions involved [186]. Recently, an association of epigenetic, DNA methylation-based, clocks in brain tissue with brain pathologies and common aging phenotypes has been demonstrated [187]. It has also been suggested that these clocks may play an important role in age prediction to age reversion [188]. Since antioxidants seem to cause adaptive epigenetic changes (in part by reducing oxidative stress), their role in age prediction to age reversion is even more plausible [189].

In conclusion, the manipulation of endogenous cellular defense mechanisms through nutritional antioxidants or pharmacological compounds represents an innovative approach to therapeutic intervention in diseases causing tissue damage, such as neurodegeneration. Consistent with this notion, antioxidants may delay and/or modulate the cognitive brain aging processes and decrease the occurrence of their devastating effects on the brain. All compounds aiming to influence brain aging must be able to surpass the limitation represented by the BBB after systemic administration.

The potential preventive activity of antioxidants should be further evaluated in long-term exposure clinical trials, using preparations with high bioavailability and that are well standardized.

## Figures and Tables

**Table 1:** Evidence on neuroprotective and cognitive role of resveratrol and hydroxytyrosol. AMPK, AMP-activated protein kinase; βA, beta amyloid, COX, cyclooxygenase; CSF, cerebrospinal fluid; ERβ, estrogen receptor β; ERK, extracellular signal-regulated kinase; LPS, lipopolysaccharide; MMP9, matrix metalloproteinase 9; NF-κB, nuclear factor κB; NO, nitric oxide, PGES-1, prostaglandin E synthase-1; TNF, tumor necrosis factor.

Study Type	Subject	Component	Dose	Main Findings	Ref.
Neuroinflammation					
Retrospective study	38 patients with Alzheimer disease and CSF Aβ42 < 600 ng/mL	Resveratrol	500 mg	Reduction of CSF MMP9, modulation of neuroinflammation, and induction of adaptive immunity.	[109]
Animal study	Rat model of Alzheimer’s disease	Resveratrol-Selenium nanoparticles	Not clear	Reduced neuroinflammation and neurotoxicity by regulating Sirt1/miRNA-134/GSK3β expression	[110]
Animal study	Adult Sprague-Dawley rats: 6-OHDA-induced Parkinson’s disease rat model.	Resveratrol	10–40 mg/kg per day for 10 weeks	Alleviation of 6-OHDA-induced chromatin condensation, mitochondrial tumefaction, and vacuolization of dopaminergic neurons in rat substantia nigra. Reduction of the reduced inflammatory reaction by lowering levels of COX-2 and TNF-α mRNA in the substantia nigra.	[111]
In vitro animal study	Primary microglial cell cultures prepared from cerebral cortices of neonatal rats	Resveratrol	1–50 μM	Reduction of microglial activation.Resveratrol is the first known inhibitor, which specifically prevents PGES-1 expression without affecting cyclooxygenase-2 (COX-2) levels.	[112]
In vitro animal study	Rat astroglioma C6 cells	Resveratrol	up to 50 μM	Reduction of microglial pro-inflammatory responses by modulation of PG, NO, and NF-κB activity	[113]
In vitro animal study	Rat astroglioma C6 cells	Resveratrol	200 μM	Modulation of NF-κB	[114]
In vitro animal study	Rat astroglioma C6 cells	21 resveratrol derivatives	Variable	3 derivatives demonstrated to be able to reduce of microglial pro-inflammatory activity by modulating TNF-α and NO synthase expression	[115]
In vitro animal study	Primary cortical neuron-glia cultures of female Wistar rats	Resveratrol	15–60 μM	Inhibition of LPS-induced microglial activation and subsequent production of multiple pro-inflammatory and cytotoxic factors (TNF-α, NO, and interleukin-1β)	[116]
In vitro	BV-2 cells	Resveratrol	0–1000 nM	Neuroprotection against hypoxia-induced neurotoxicity through inhibiting microglial activation by suppressing the activation of NF-ĸB, ERK, and JNK-MAPK signaling pathways	[117]
In vitro	Vascular adventitial fibroblasts isolated from rats	Hydroxytyrosol	12.5, 25, 50, 100, 200, 400 μM	Regulation of the autophagy of vascular adventitial fibroblasts through SIRT1-mediated Akt/mTOR suppression.Inhibition of the inflammatory response of vascular adventitial fibroblasts	[118]
In vitro and ex vivo	Hypoxia-reoxygenation in rat brain slices	Hydroxytyrosol	1, 5 and 10 mg/kg per day	Neuroprotective effect due to antioxidant and anti-inflammatory activity	[119]
In vitro and ex vivo	Hypoxia-reoxygenation model in rat brain slices	Hydroxytyrosol derivatives	Variable	Neuroprotective effect due to reduction in oxidative and nitrosative stress and anti-inflammatory activity.Reduction in brain cell death.	[120]
Experimental animal study	APP/PS1 transgenic mice	Hydroxytyrosol	5 mg/kg/day	Ameliorated mitochondrial dysfunction, reduced mitochondrial carbonyl protein, and enhanced superoxide dismutase 2 expression, reversed the phase 2 enzyme system and reduced the levels of the brain inflammatory markers	[121]
Oxidative stress					
In vitro	HepG2 cells	Resveratrol	10–60 μM	Protection of mitochondria against oxidative stress through AMPK-mediated glycogen synthase kinase-3β inhibition	[122]
In vitro	Rat hippocampal cells	Resveratrol	5–25 μM	Protection of hippocampal neuronal cells against toxicity induced by NO	[123]
Experimental animal study	*Caenorhabditis elegans*	Hydroxytyrosol	100 μg/mL	Prevention of oxidative stress and β-amyloid aggregation	[124]
In vitro and ex vivo	Type-1-like diabetic hypoxia-reoxygenation model in brain slices	3’,4’-dihydroxyphenylglycol and hydroxytyrosol	5 mg/kg/day (hydroxytyrosol) and 0.5 or 1 mg/kg/day (3′,4′-dihydroxyphenylglycol)	A 1:1 ratio of hydroxytyrosol/3’,4’-dihydroxyphenylglycol results in reduced brain cell death, neuroprotective, and antioxidant effects	[125]
Animal study	Wistar rats	Hydroxytyrosol	2.5 mg/kg per day	Brain protection against the oxidative stress caused by 3-nitropropionic acid	[126]
Cerebrovascular function					
Randomized, double-blinded clinical trial	22 healthy adults	Resveratrol	250 and 500 mg	Increases cerebral blood flow during cognitive task performance in health adults but lacking interpretable cognitive effects	[127]
Randomized, double-blinded clinical trial	60 adults	Resveratrol	500 mg	Increases cerebral blood flow but lacking interpretable cognitive effects	[128]
Randomized clinical trial	80 post-menopausal women	Resveratrol	75 mg twice daily	Enhance both cerebrovascular function and cognition in post-menopausal women	[129]
Randomized, double-blinded clinical trial	125 postmenopausal women	Resveratrol	75 mg twice daily	Enhance cognition, cerebrovascular function, and insulin sensitivity	[130]
Randomized, double-blinded clinical trial	129 postmenopausal women	Resveratrol	75 mg twice daily	Enhance cognition and cerebrovascular function	[131]
Randomized, double-blinded clinical trial	36 dementia-free, non-insulin dependent type 2 diabetes mellitus adults	Resveratrol	0, 75, 150, and 300 mg at weekly intervals	Acute enhancement of vasodilator responsiveness in cerebral vessels.The maximum improvement was observed with the lowest dose used.	[132]
Autophagy					
Animal study	Rats with chronic cerebral hypoperfusion	Resveratrol	50 mg/kg per day	Autophagy activation via the AKT/mTOR signaling pathway to improve cognitive dysfunction.	[133]
Animal study	120 Sprague-Dawley rats	Resveratrol	60 mg/kg	Neuroprotective effects due to regulating autophagy and apoptosis mediated by the Akt/mTOR pathway	[134]
Animal study	Rats	Resveratrol	1.8 mg/Kg	Neuroprotective effects due to regulating autophagy through AMPK	[135]
Neuroprotective effect enhancement					
Randomized, double-blinded clinical trial	23 adults	Resveratrol andpiperine	250 mg (resveratrol), 20 mg (piperine)	Co-supplementation of piperine with resveratrol enhances the effects of resveratrol on cerebral blood flow effects without altering bioavailability.	[136]
In vitro animal study	Murine HT22 hippocampal cells	Resveratrol and melatonin	Resveratrol: 0.1, 1, 5, 10, and 20 µM.Melatonin: 1, 10, 50, 100, and 500 µM.	Melatonin potentiates the neuroprotective properties of resveratrol against Aβ-induced toxicity by modulating GSK3β and AMPK activity	[137]
Detoxification					
In vitro/in vivo animal study	Primary hippocampal cell cultures from pregnant Sprague–Dawley rats	Resveratrol	15–40 µM	Neuroprotection against βA-induced neurotoxicity by inducing the phosphorylation of protein kinase Cδ isoform	[138]
In vitro	C12 cells	Hydroxytyrosol	Hydroxytyrosol -rich extract based with 45.5% of hydroxytyrosol	Brain cell cryoprotection	[139]
Animal study	Piglets	Hydroxytyrosol	1.5 mg/kg per day	Upregulation of proteins related to brain cell detoxification.	[140]
Cognitive impairment					
Randomized double-blinded controlled trial	119 patients with mild to moderate Alzheimer disease	Resveratrol	500 mg	Resveratrol is well tolerated and seems to be able to penetrate the blood–brain barrier to produce its neuroprotective effects	[141]
Animal study	Mice	Resveratrol	5 and 10 mg/kg	Protection from 3-nitropropionic acid-induced motor and cognitive impairment	[142]
In vitro/in vivo animal study	Hippocampal slice cultures from Sprague–Dawley rats exposed to ischemia	Resveratrol	75 and 100 μM	Reduction of neuronal death in CA1 region of the hippocampus by activation of SIRT1 pathway	[143]
Experimental animal study	APP/PS1 transgenic mice	Hydroxytyrosol	5 mg/kg/day	Improves the cognitive function in ERβ-dependent manner	[144]
Animal study	C57BL/6 mice	Hydroxytyrosol	10 mg/kg per day	Attenuation of the spatial-cognitive deficits induced by oligomeric Aβ1–42 plus ibotenic acid	[145]
Animal study	Sprague–Dawley rats	Hydroxytyrosol	10 and 50 mg/kg per day	Restoration of learning capacity and memory performance, promoting cognitive function	[146]
Animal study	wild-type and B-cell translocation 1 gene knockout mice	Hydroxytyrosol	100 mg/kg/day	Stimulates neurogenesis in aged dentate gyrus by enhancing stem and progenitor cell proliferation and neuron survival	[147]
In vitro and ex vivo	Hypoxia-reoxygenation in rat brain slices	Hydroxytyrosol	5 or 10 mg/kg per day	Reduction in brain cell death	[148]
Animal study	Piglets	Hydroxytyrosol	1.5 mg/kg per day	Maternal supplementation with hydroxytyrosol during pregnancy affects the neurotransmitters profile in a brain-area-dependent mode and accelerates cell differentiation in the hippocampal CA1 and GD areas.	[149]

## Data Availability

Not applicable.
